# Examining the Factor Structure of the 39-Item and 15-Item Versions of the Five Facet Mindfulness Questionnaire Before and After Mindfulness-Based Cognitive Therapy for People With Recurrent Depression

**DOI:** 10.1037/pas0000263

**Published:** 2016-04-14

**Authors:** Jenny Gu, Clara Strauss, Catherine Crane, Thorsten Barnhofer, Anke Karl, Kate Cavanagh, Willem Kuyken

**Affiliations:** 1 School of Psychology, University of Sussex, and Sussex Partnership NHS Foundation Trust, Hove, England; 2 Department of Psychiatry, University of Oxford, Warneford Hospital, Oxford, England; 3 Dahlem Center for Neuroimaging of Emotion, Freie Universitaet Berlin; 4 Psychology, College of Life and Environmental Sciences, University of Exeter; 5 School of Psychology, University of Sussex; 6 Department of Psychiatry, University of Oxford, Warneford Hospital, Oxford, England

**Keywords:** mindfulness, MBCT, FFMQ, confirmatory factor analysis, configural invariance

## Abstract

Research into the effectiveness and mechanisms of mindfulness-based interventions (MBIs) requires reliable and valid measures of mindfulness. The 39-item Five Facet Mindfulness Questionnaire (FFMQ-39) is a measure of mindfulness commonly used to assess change before and after MBIs. However, the stability and invariance of the FFMQ factor structure have not yet been tested before and after an MBI; pre to post comparisons may not be valid if the structure changes over this period. Our primary aim was to examine the factor structure of the FFMQ-39 before and after mindfulness-based cognitive therapy (MBCT) in adults with recurrent depression in remission using confirmatory factor analysis (CFA). Additionally, we examined whether the factor structure of the 15-item version (FFMQ-15) was consistent with that of the FFMQ-39, and whether it was stable over MBCT. Our secondary aim was to assess the general psychometric properties of both versions. CFAs showed that pre-MBCT, a 4-factor hierarchical model (excluding the “observing” facet) best fit the FFMQ-39 and FFMQ-15 data, whereas post-MBCT, a 5-factor hierarchical model best fit the data for both versions. Configural invariance across the time points was not supported for both versions. Internal consistency and sensitivity to change were adequate for both versions. Both FFMQ versions did not differ significantly from each other in terms of convergent validity. Researchers should consider excluding the Observing subscale from comparisons of total scale/subscale scores before and after mindfulness interventions. Current findings support the use of the FFMQ-15 as an alternative measure in research where briefer forms are needed.

Mindfulness is commonly defined as “paying attention in a particular way; on purpose, in the present moment, and non-judgmentally” (Kabat-Zinn, 1994, p. 4). It involves being aware and accepting of internal and external moment-to-moment experience and relating to thoughts and emotions in a decentered manner as “mental events,” rather than accurate reflections of the self and reality. Mindfulness is regarded as a universal human capacity that can enhance well-being (e.g., Ludwig & Kabat-Zinn, 2008). The secular practice of mindfulness has been integrated into various clinical interventions, with a view to increasing mindfulness and, as a consequence, improving mental health and well-being. The two most extensively applied and assessed mindfulness-based interventions (MBIs) are mindfulness-based stress reduction (MBSR; Kabat-Zinn, 1982) and mindfulness-based cognitive therapy (MBCT; Segal, Williams, & Teasdale, 2002, 2013). Both MBSR and MBCT are eight-session group-based programs in which participants engage in formal and informal mindfulness meditation practices during sessions and at home. MBSR was developed to alleviate distress, pain, stress, and anxiety in people with chronic physical health problems through the cultivation of mindfulness. More recently, MBCT was designed for people with recurrent major depressive disorder (MDD) in remission as a relapse prevention intervention. MBCT is theorized to decrease depressive recurrence by enhancing mindful awareness of and disengagement from dysphoria-triggered repetitive negative thinking (e.g., rumination) about one’s depressive symptoms (Segal et al., 2002, 2013).

Meta-analytic reviews have found MBCT and MBSR to be effective in improving a range of outcomes in clinical and nonclinical samples including stress, depression, depressive relapse, and anxiety (e.g., Chiesa & Serretti, 2009; Hofmann, Sawyer, Witt, & Oh, 2010; Piet & Hougaard, 2011; Strauss, Cavanagh, Oliver, & Pettman, 2014). As evidence for the effectiveness of MBIs is accumulating and these interventions are being adapted to target a broad range of problems, there is an increasing need for investigations of the mechanisms of change (see Gu, Strauss, Bond, & Cavanagh, 2015, for a review). At the very basis of this endeavor is whether MBIs work through their purported mechanisms of action (e.g., by increasing mindfulness) and which aspects of the construct of mindfulness are being affected by the training.

Psychological outcomes and processes from effectiveness and mechanism studies have been predominantly measured using self-report questionnaires, due to their cost-effectiveness and standardized, easy-to-administer format. Among the available self-report measures of mindfulness, the Five Facet Mindfulness Questionnaire (FFMQ; Baer, Smith, Hopkins, Krietemeyer, & Toney, 2006) is a widely used measure that aims to capture the key underlying dimensions of mindfulness (Sauer et al., 2013).

## The Five Facet Mindfulness Questionnaire

The FFMQ is a 39-item (FFMQ-39) self-report measure of the dispositional tendency to be mindful in daily life. The questionnaire derived from an exploratory factor analysis (EFA; Baer et al., 2006) of items from five independently developed self-report mindfulness scales: the (a) Mindfulness Attention Awareness Scale (Brown & Ryan, 2003), (b) Freiburg Mindfulness Inventory (Walach, Buchheld, Buttenmuller, Kleinknecht, & Schmidt, 2006), (c) Cognitive Affective Mindfulness Scale (A. M. Hayes & Feldman, 2004), (d) Mindfulness Questionnaire (Chadwick, Taylor, & Abba, 2005), and (e) Kentucky Inventory of Mindfulness Skills (Baer, Smith, & Allen, 2004). Baer et al.’s (2006) findings showed that mindfulness can be conceptualized as a multifaceted construct consisting of five related dimensions: Observing, Describing, Acting with Awareness, Nonjudging of Inner Experience, and Nonreactivity to Inner Experience. Observing refers to attending or noticing internal and external experiences (e.g., sounds, emotions, thoughts, bodily sensations, smells). Describing includes the ability to express in words one’s experiences. Acting with awareness involves attending to one’s present moment activity, rather than being on “autopilot,” or behaving automatically, while attention is focused elsewhere. Nonjudging of inner experience involves accepting and not evaluating thoughts and emotions (e.g., as “good” or “bad”). Finally, nonreactivity to inner experience refers to the ability to detach from thoughts and emotions, allowing them to come and go without getting involved or carried away by them. The 39 items of the FFMQ are rated on a 5-point Likert scale, ranging from 1 (*never or very rarely true*) to 5 (*very often or always true*). In addition to considering scores on the five subscales individually, facet scores can be combined to produce an overall mindfulness score.

Analyses of the psychometric properties of the FFMQ-39 have generally demonstrated that this measure has satisfactory convergent and discriminant validity, internal consistency, interpretability in distinguishing between participant subgroups, and incremental validity in predicting psychological symptoms and well-being across samples of regular meditators and nonmeditators (students, general community sample, adults with heterogeneous mood and anxiety disorders, adults with moderate depression or anxiety symptoms; e.g., Baer et al., 2006; Baer et al., 2008; Bohlmeijer, ten Klooster, Fledderus, Veehof, & Baer, 2011; Christopher, Neuser, Michael, & Baitmangalkar, 2012; Curtiss & Klemanski, 2014a, 2014b). The FFMQ-39 has also been shown to have good sensitivity to change; researchers have found moderate-to-large, and statistically significant, increases in all five facets before and after MBSR in a sample of adults with chronic pain and heterogeneous mood and anxiety disorders (Carmody & Baer, 2008) and before and after a 9-week therapeutic intervention based on mindfulness in a sample of adults with mild-to-moderate symptoms of depression or anxiety (Bohlmeijer et al., 2011).

Although the psychometric properties of the FFMQ-39 have been supported, findings from a series of confirmatory factor analysis (CFA) studies question the validity of its five-factor structure and the inclusion of all five subscales in MBI research. The five-factor structure emerging in the development of the FFMQ-39 using EFA (Baer et al., 2006) has been confirmed in meditator samples, in which a five-factor hierarchical model (with the five related factors subsumed under an overarching mindfulness construct) provided the optimal fit for the data (Baer et al., 2008; M. J. Williams, Dalgleish, Karl, & Kuyken, 2014). However, for nonmeditator samples (general community sample, students, adults with recurrent MDD in remission, adults with heterogeneous mood and anxiety disorders), a four-factor hierarchical model (with all facets minus observing loading onto an overall mindfulness factor) best fit the data (Baer et al., 2006; Baer et al., 2008; Curtiss & Klemanski, 2014a; M. J. Williams et al., 2014). Poor fit of the five-factor hierarchical model in nonmeditator samples can be attributed to the nonsignificant relations found between observing and nonjudging (Baer et al., 2006; Bohlmeijer et al., 2011; Curtiss & Klemanski, 2014a), and observing and acting with awareness (Curtiss & Klemanski, 2014a).

A possible explanation for these differing factor structures across meditators and nonmeditators is that the qualities of observing may differ in meditators and nonmeditators, such that increased meditation practice strengthens the relations between observing and the other mindfulness facets (Baer et al., 2008). For nonmeditators, observing items (e.g., “When I’m walking, I deliberately notice the sensations of my body moving”) may be equally likely to reflect neutral attention, or even maladaptive, biased, and pathological forms of attention (e.g., anxious monitoring, hypervigilance to threat), rather than attention characterized by the curious, accepting, and purposeful quality cultivated through mindfulness meditation practice. Therefore, people with little or no mindfulness experience may report how much they tend to observe, but the way in which they notice may or may not be related to mindful qualities assessed by the other facets, resulting in the emergence of a four-factor hierarchical solution. By contrast, people with meditation experience may respond to observing items in a way that is more consistent with the other four facets and with a mindful disposition, thus resulting in the emergence of a five-factor hierarchical solution. In support of this explanation, findings have shown that, in nonmeditators (student, community, and highly educated samples), observing was the only facet that correlated positively with psychological symptoms; in meditators, all facets correlated negatively with psychological symptoms (Baer et al., 2008).

To reduce participant burden in research trials, which include multiple measures and/or measures administered on multiple occasions, short versions of the original FFMQ-39 have been developed. One such version is a 24-item FFMQ, which has been shown to replicate the five-factor structure of the original measure, to be highly correlated with the original version, and to be sensitive to change (Bohlmeijer et al., 2011). More recently, a 15-item version (FFMQ-15) has been developed, which includes three items for each of the five facets (Baer, Carmody, & Hunsinger, 2012). These items were selected based on the factor loadings for each subscale of the FFMQ-39 (Baer et al., 2006) and to maintain the breadth of content for each facet. However, the factor structure, correlation with the FFMQ-39, convergent validity, and sensitivity to change of the FFMQ-15 have not yet been validated.

Findings that highlight different factor structures for the FFMQ-39 in meditators and nonmeditators (Baer et al., 2006; Baer et al., 2008; Curtiss & Klemanski, 2014a; M. J. Williams et al., 2014) have implications for studies using this measure to compare levels of mindfulness across these two samples or to evaluate the effectiveness of MBIs in samples with no previous meditation experience. Although the factor structure of the FFMQ-39 has been tested in a number of samples (e.g., meditators, students, general community sample, adults with recurrent MDD in remission, adults with heterogeneous mood and anxiety disorders), no known studies have yet directly examined the stability of the factor structure before and after mindfulness training (e.g., through MBCT) in a single sample. Previous studies have suggested that meditation status results in differential factor structures emerging for the FFMQ-39; a study evaluating the measure’s factor structure before and after an MBI in the same sample would provide a stronger test of whether mindfulness meditation experience changes the factor structure of the FFMQ-39.

## The Present Study

The primary aim of this study was to examine the stability of the factor structure of the FFMQ-39 before and after MBCT using CFA. Because the FFMQ-15 has not yet been validated, we also examined whether its factor structure was consistent with that of the original version, and whether the factor structure of the FFMQ-15 was invariant over a period in which people were learning mindfulness through MBCT. Data from two trials evaluating MBCT for adults with MDD in remission were used, meaning that the people contributing data were representative of the population for whom MBCT was originally designed. The secondary aim of this study was to assess the general psychometric properties of the FFMQ-39 and FFMQ-15. Each facet’s sensitivity to change over the course of MBCT was examined. Convergent validity of the FFMQ-39 and FFMQ-15 were also tested by correlating the facets with theoretically related constructs before and after MBCT, specifically, measures of depression and negative rumination. Significant moderate negative correlations were expected between rumination and depression and the facets describing, acting with awareness, nonjudging, and nonreactivity of both versions of the FFMQ. Given that research into how meditation experience might alter the way in which people observe is still emerging, no hypotheses were made about the correlations between the observing facet of the FFMQ-39 and FFMQ-15 and rumination and depression.

## Method

### Participants and Design

The sample consisted of participants from two trials that examined the effectiveness of MBCT compared with control conditions at reducing relapse into depression for people with recurrent MDD in remission (Preventing depressive relapse in NHS settings through MBCT [PREVENT] trial; Kuyken et al., 2015; and Staying Well After Depression [SWAD] trial; J. M. G. Williams et al., 2014). M. J. Williams et al.’s (2014) CFA study also used data from PREVENT to examine the factor structure of the FFMQ-39 at baseline. However, our study used an extended sample and differed from theirs in the research questions tested; M. J. Williams et al. compared the FFMQ factor structure across independent samples of meditators and nonmeditators at one time point, whereas we examined the stability of the FFMQ structure before and after MBCT in a single sample.

Both PREVENT and SWAD were multicenter trials, with PREVENT recruiting from general practices in rural and urban settings in the United Kingdom and SWAD recruiting from the community, primary care, and mental health clinics in the regions of Oxford, England, and Bangor, North Wales. Inclusion criteria for both trials were: (1) a diagnosis of recurrent MDD in full or partial remission according to the *Diagnostic and Statistical Manual of Mental Disorders−IV* (American Psychiatric Association, 1994), (2) three or more previous depressive episodes, and (3) being 18 years or older. Exclusion criteria from both trials were: having (a) a current major depressive episode, (b) a comorbid diagnosis of current substance misuse, organic brain damage, current or past psychosis, current or past bipolar disorder, persistent antisocial behavior, or persistent self-harm requiring clinical management or therapy, and (c) formal concurrent psychotherapy. Only data from participants in the MBCT arm of both trials, who completed all FFMQ items both before and after MBCT and who took part in at least four of eight sessions of MBCT (i.e., who were deemed therapy completers; Teasdale et al., 2000), were used in this study.

The total number of participants who fit the criteria was 238 (74.38% of the total number of participants randomized to MBCT in PREVENT and SWAD), 154 participants from the PREVENT trial and 84 participants from the SWAD trial. Of the 238 participants, 69 (29%) were men and 169 (71%) were women. Mean age of the sample was 49.18 years (*SD* = 12.01; range: 23–78 years). Most (97.5%) of the sample was white. In terms of educational qualifications, 13 (5.55%) had no qualifications, 34 (14.3%) had some General Certificate of Secondary Education/O Levels, 71 (29.8%) had some A Levels or comparable vocational qualifications, 57 (23.9%) had a bachelor’s degree, 24 (10.1%) had a master’s degree, and 30 (21.6%) had a doctoral degree or professional qualification. Three participants had other qualifications and data on education were missing for six.

### Measures

#### Five Facet Mindfulness Questionnaire

The 39-item FFMQ (Baer et al., 2006) measures the trait-like tendency to be mindful in daily life. It is comprised of the following five related facets: observing, describing, acting with awareness, nonjudging, and nonreactivity. Sample items include: “I notice the smells and aromas of things” (observing), “I’m good at finding words to describe my feelings” (describing), “I find myself doing things without paying attention” (acting with awareness), “I disapprove of myself when I have illogical ideas” (nonjudging), and “When I have distressing thoughts or images, I do not let myself be carried away by them” (nonreactivity). Facet scores range from 8−40, with the exception of the nonreactivity facet, which ranges from 7−35. The 15-item FFMQ (Baer et al., 2012) includes the following items of the FFMQ-39 for each of the five facets: Items 6, 11, and 15 for observing, Items 2, 16, and 27 for describing, Items 8, 34, and 38 for acting with awareness, Items 10, 14, and 30 for nonjudging, and Items 19, 29, and 33 for nonreactivity. These items were selected by Baer et al. (2012) based on their factor loadings and to maintain the range of content for each facet. The FFMQ-15 is measured using the same scale as the FFMQ-39 and its facet scores range from 3−15. In the current study, only the FFMQ-39 was administered to participants; FFMQ-15 data were extracted from the 39-item version. Cronbach’s alphas for facets from both versions of the measure are displayed in Table 1.[Table-anchor tbl1]

#### Beck Depression Inventory−II

The 21-item Beck Depression Inventory−II (BDI-II; Beck, Steer, & Brown, 1996) is widely used to assess the severity of depressive symptomatology. Each item is a list of four statements about a symptom of depression, arranged in order of severity. Items are rated on a 4-point scale ranging from 0 (*not at all*) to 3 (*extreme form of each symptom*), which corresponds to each statement. Items are summed to give a single total score, which ranges from 0−63; a score of 0–13 is considered to reflect minimal depression, 14–19 mild depression, 20–29 moderate depression, and 30–63 severe depression. Cronbach’s alpha was .93 for this sample at both baseline and post-MBCT.

#### Cambridge Exeter Ruminative Thinking Scale

The Cambridge Exeter Ruminative Thinking Scale (CERTS; Barnard, Watkins, Mackintosh, & Nimmo-Smith, 2007) is a transdiagnostic tool for assessing multiple aspects of rumination. The measure consists of three parts, each with several subscales. The first scale measures patterns of ruminative thinking across multiple contexts (e.g., anxious, happy, sad) and consists of two subscales: rumination in response to negative mood and negative exigencies (Negative Rumination) and rumination in response to positive mood and progress (Positive Rumination). The second part assesses the products and consequences of rumination, or whether respondents view their ruminative thinking as helpful or not, and consists of three subscales: Constructive Resolution, Ongoing Uunresolution, and Move On/Put Behind Me. The third part examines the processes of ruminative thinking and includes four subscales: Comparative Negative Rumination or Affective Interlock, Expansive/Dendritic Thinking, Analytic−Evaluative Abstract Thinking, and Rapid Non-Analytic/Experiential Thinking. Because MBCT theory highlights repetitive negative thinking as a key mechanism underlying the intervention’s effects (Segal et al., 2002, 2013), we only used the CERTS Negative Rumination subscale. This subscale consists of 20 items measuring the frequency, duration, controllability, and repetition of rumination in response to five negative contexts (when I feel sad/angry/anxious, when I am by myself, and when I experience a setback on something I value). Items are rated on a 4-point Likert scale ranging from 1 (*almost never*) to 4 (*almost always*), with subscale scores ranging from 20−80. Total scores provide a general index of the severity of rumination, with higher scores indicating greater negative rumination. In the current sample, only participants from the PREVENT trial (*n* = 154) completed this measure. Cronbach’s alphas for the Negative Rumination subscale in this sample at baseline and post-MBCT were .82 and .83, respectively.

### Procedure

Participants completed the FFMQ as well as other measures both before and after MBCT. Measures were administered by research assistants blind to group allocation. The MBCT program integrates intensive mindfulness meditation practice with psychological education from cognitive–behavioral therapy for depression (Segal et al., 2002, 2013). The program in both the PREVENT and the SWAD trials consisted of eight weekly 2- to 2.25-hr group sessions and followed the manualized MBCT intervention described by Segal et al. The groups were delivered by therapists who had met the Mindfulness-Based Interventions Teacher Assessment Criteria (Crane et al., 2013), to ensure that the sessions were delivered to a high standard and adhered to the MBCT manual. Written informed consent was obtained from all participants.

### Statistical Analyses

#### Preliminary analyses

Preliminary analyses were conducted to check for univariate and multivariate normality, and to report the descriptive statistics and general psychometric properties of the FFMQ-39 and FFMQ-15. Cronbach’s alpha reliability coefficients were computed for subscales from both versions of the FFMQ. Sensitivity to change pre- to post-MBCT was also examined for FFMQ-39 and FFMQ-15 facets using paired-samples *t* tests and accompanying Cohen’s *d* effect sizes and 95% confidence intervals (CIs) for *d*, calculated using Equations 4, 15, and 18 from Nakagawa and Cuthill (2007):

$$d=t_{\text{paired}}\;\sqrt{\frac{2(1\;-\;r_{12})}{n}}\;$$ 4

95% CI = ES−1.96se⁢ to ES + 1.96se 15

$$se_{d}=\;\sqrt{\frac{2\left(1-\;r_{12}\right)}{n}+\frac{d^{2}}{2(n-1)}}$$ 18

where *d* and *ES* are Cohen’s *d* effect size; *t*_paired_ is the *t* value from the paired *t* test; *r*_12_ is the correlation coefficient between the two groups; *n* = *n*_1_ = *n*_*2*_; CI is the confidence interval; *se* is the asymptotic standard error for *d*.

Pearson correlation coefficients (*r*) were conducted to examine the relations between the FFMQ-39 and FFMQ-15 facets at baseline. Because data for both long and short versions of the FFMQ were based on a single administration of the measure, we additionally computed Levy’s (1967) corrected correlation coefficients (*r*_c_) to adjust for overlapping error variance and spuriously inflated correlations between the long and short versions. Levy’s corrected correlations were calculated using the Shortform Version 1.1 software developed by Barrett (2005).

Pearson correlations were also calculated between FFMQ-39 and FFMQ-15 total facet scores and total scores on the BDI-II and the CERTS Negative Rumination subscale before and after MBCT to examine convergent validity. To determine whether correlation coefficients with the BDI-II and CERTS differed in size for both versions of the FFMQ, Steiger’s (1980) *z* tests were conducted. This test is recommended for comparing two correlations with one variable in common from the same sample (Meng, Rosenthal, & Rubin, 1992). These tests determined whether there were statistically significant differences between FFMQ-39 and FFMQ-15 facets in their correlations with the BDI-II and CERTS before and after MBCT (e.g., whether there was a significant difference between the correlation coefficient for FFMQ-39 describing and BDI-II pre-MBCT and the correlation for FFMQ-15 describing and BDI-II pre-MBCT). Steiger’s *z* tests were conducted using software developed by Lee and Preacher (2013). Because this test was conducted 20 times (comparing each of the five facet’s correlation with BDI-II and CERTS at baseline and post-MBCT across the two versions), Bonferroni-corrected alpha levels of *p* equal to .0025 (.05/20) were used. Excluding those conducted using the Shortform Version 1.1 and Steiger’s *z* software, all preliminary analyses were carried out with SPSS Amos, Version 22 (IBM, 2013).

#### Confirmatory factor analyses

To replicate Baer et al. (2006) and M. J. Williams et al. (2014), the following five models were tested separately for both the short and the long versions of the FFMQ before and after MBCT using CFA: (a) a one-factor model in which all items were indicators of an overall, latent mindfulness factor, (b) a five-factor model in which items were indicators of their respective five correlated mindfulness factors, (c) a five-factor hierarchical model in which the five factors were indicators of an overarching mindfulness factor, (d) a four-factor hierarchical model in which four factors (minus the observing facet) were indicators of an overarching mindfulness factor, and (e) a four-factor model in which items were indicators of their respective four correlated mindfulness factors (minus the observing facet). To replicate the procedure used by Baer et al. (2006; Baer et al., 2008) and M. J. Williams et al. (2014), the CFAs of the 39-item FFMQ were conducted using item parcels (groups of items) rather than individual items.

Following Baer et al. (2008), within each facet, items were allocated sequentially to parcels in the order that they appear on the FFMQ (i.e., first item to Parcel 1, second to Parcel 2, etc.) and item scores within each parcel were averaged. A total of 15 parcels (three parcels per facet, with two or three items per parcel) were used for the CFAs of the FFMQ-39. Item parceling is a controversial practice with several advantages and disadvantages (see Little, Cunningham, Shahar, & Widaman, 2002, for a discussion of the strengths and limitations of parceling). One strength of parceling is that the reliability and the stability of a parcel as an indicator of a latent construct tend to be greater than those of an individual item. However, opponents of parceling have argued that parcels can obscure model misspecifications, by improving model fit whether or not the model is correctly specified. Considering both the pros and cons of parceling, Little et al. concluded that this technique can be particularly effective when items within parcels are unidimensional, or measuring the same construct. Other researchers have also stated that parceling should be considered only when there is unidimensionality (e.g., Bandalos & Finney, 2001). In the case of the FFMQ, unidimensionality of its items has been established using EFA (Baer et al., 2006). Thus, parceling was deemed appropriate for the current study. Because the 15-item FFMQ consists of just three items per facet, which would not be feasible to parcel, the individual items were used in the CFAs for this version. In all separate CFA models, error terms were not allowed to covary and items or parcels were constrained to load onto only one factor.

The following six fit indices were used collectively to indicate the global fit of the models to the data: the comparative fit index (CFI; Bentler, 1990), the root mean square error of approximation (RMSEA; Steiger, 1990), the non-normed fit index (NNFI; Bentler & Bonett, 1980), the standardized root mean square residual (SRMR), the chi-square model test, and the Akaike information criterion (AIC; Akaike, 1974). Rules of thumb for the cutoff values that indicate acceptable index fit are as follows: the CFI and NNFI should be .95 or higher (Hu & Bentler, 1999), the RMSEA should be less than .10 (Browne & Cudeck, 1993), and the SRMR should be less than .10 (Hu & Bentler, 1999). The AIC was used as a measure of model parsimony to compare the fit of the five models; the lower the value, the better the fit. The chi-square test is generally not recommended to evaluate model fit because of its sensitivity to non-normality, large correlations between variables, large sample sizes, and variables with high proportions of unique variance (Kline, 2011). Therefore, we reported the chi-square test alongside alternative fit indices, but did not use it as a primary measure of model fit.

Schermelleh-Engel, Moosbrugger, and Müller (2003) cautioned that cutoff criteria can be arbitrary, such that a model may provide a good fit to the data even when one or more fit indices suggest poor fit, and vice versa. Therefore, based on the systematic procedure used by M. J. Williams et al. (2014), the following criteria were also considered when interpreting which model provided a superior fit to the data: (a) significant loadings of items, parcels, or facets onto relevant latent factors, (b) significant covariances between facets, and (c) lowest AIC. All CFAs were conducted in SPSS Amos, Version 22.

#### Factorial invariance

In addition to the separate CFA models conducted for both versions of the FFMQ before and after MBCT, we evaluated longitudinal factorial invariance, or whether the same construct is assessed across time, using the analytic approach by Widaman, Ferrer, and Conger (2010). Widaman et al. described four levels involved in establishing factorial invariance. The first step involves testing for configural invariance of the five-factor hierarchical models before and after MBCT simultaneously in a single model. Two longitudinal configural invariance models were tested; one for the FFMQ-39 and one for the FFMQ-15. These tests aimed to establish whether the structural configuration (number of factors and pattern of factor loadings) of the FFMQ was equal across the time points. Good global model data fit would indicate that the structural configuration of the FFMQ remains stable before and after MBCT. Poor fit of a longitudinal configural model would indicate that the five-factor hierarchical structure of the FFMQ does not apply both before and after MBCT. Covariances were included between the overarching mindfulness factors pre- and post-MBCT, and between the pre-MBCT items/parcels and the corresponding items/parcels post-MBCT. Minimum identification constraints were placed on parameters.

Once configural invariance has been established, the next steps would be to test for weak factorial invariance (invariant factor loadings across time), then strong factorial invariance (invariant factor loadings and intercepts across time), and finally strict factorial invariance (invariant factor loadings, intercepts, and factor variances across time; Widaman et al., 2010). This sequence involves gradually increasing the constraints placed on the model parameters. For the FFMQ-39 and FFMQ-15 to be measuring the same construct before and after MBCT, strong or strict factorial invariance must be met. If a preceding level of factorial invariance was not supported, we did not proceed to establish the next level(s) by applying further model constraints.

## Results

### Preliminary Analyses

All 15 parcels of the FFMQ-39 pre- and post-MBCT were normally distributed, as assessed by checking histograms, box plots, and skewness and kurtosis values. The individual items of the FFMQ-15 pre- and post-MBCT were also normally distributed. No outliers were identified when checking the standardized values for all parcels of the FFMQ-39 and individual items of the FFMQ-15 pre- and post-MBCT. However, Mardia’s (1985) test indicated that none of the CFA models met the assumption of multivariate normality. Under non-normal conditions, the chi-square model test statistic tends to be inflated (so correctly specified models are more likely to be rejected) whereas parameter standard errors tend to be underestimated (so parameters are more likely to be significant; Chou & Bentler, 1995). Bootstrapping is an approach to managing multivariate non-normality that has been found by empirical studies to perform well relative to other approaches (e.g., Nevitt & Hancock, 2001). Bootstrapping methods in Amos adjust both the *p* value associated with the chi-square test (Bollen-Stine bootstrap method; Bollen & Stine, 1992) and parameter standard errors (90% bias-corrected CIs). Therefore, all models were analyzed twice; first, using just maximum-likelihood estimation (MLE; assumes multivariate normality) and second using bootstrapping with 2,000 samples. These two approaches yielded different findings in terms of the significance of some chi-square statistics (the Bollen-Stine chi-square *p* values for the post-MBCT FFMQ-39 and FFMQ-15 four-factor, four-factor hierarchical, and five-factor models were greater than .05; using MLE, all chi-square tests were significant). However, the chi-square model test is not typically recommended to evaluate the model data fit (Kline, 2011), and so we did not use it as a primary measure of model fit.

Descriptive statistics, Cronbach’s alpha reliability coefficients, and sensitivity to change statistics for the 39-item and 15-item FFMQ facets before and after MBCT are given in Table 1. Cronbach’s alphas for the FFMQ-39 subscales ranged from .78−.88 pre-MBCT and .82−.90 post-MBCT, which correspond closely to the values found in previous research (Baer et al., 2006; Baer et al., 2008; M. J. Williams et al., 2014). Internal consistency values for the FFMQ-15 subscales were generally lower, ranging from .64−.80 pre-MBCT and .69−.83 post-MBCT. These alpha values are consistent with the range found in previous research using the FFMQ-15 (Baer et al., 2012) and are considered adequate for measures of psychological constructs (Kline, 1999). Differences in internal consistencies between the two forms are unsurprising given that alpha increases with the number of items in a measure (Cortina, 1993). The FFMQ-39 and FFMQ-15 were found to be sensitive to change, as indicated by small/moderate to moderate/large significant increases in subscale scores from pre- to post-MBCT for both versions. Correlations between the total facet scores of the FFMQ-15 and FFMQ-39 at baseline were large and significant, indicating that both versions measured highly similar constructs: *r* = .87 for observing (*r*_c_ = .70), *r* = .94 for describing (*r*_c_ = .85), *r* = .85 for acting with awareness (*r*_c_ = .71), *r* = .90 for nonjudging (*r*_c_ = .80), and *r* = .91 for nonreactivity (*r*_c_ = .75) (*p*s < .01).

### Convergent Validity

Table 2 presents the correlations between baseline total facet scores on the FFMQ-39 and FFMQ-15 and baseline scores on other constructs, and post-MBCT FFMQ-39 and FFMQ-15 facet scores and post-MBCT scores on other measures. Significant small/moderate to large negative correlations were found between facets of the FFMQ-39 and FFMQ-15 and depression (BDI-II) and negative rumination (CERTS) at both time points. Steiger’s *z* tests showed that there were no significant differences between the FFMQ-39 and FFMQ-15 facets in their correlations with BDI-II and CERTS at both pre- and post-MBCT (e.g., there was no significant difference between the correlation coefficient for FFMQ-39 acting with awareness and BDI-II at baseline [*r* = −.38] and the correlation for FFMQ-15 acting with awareness and BDI-II at baseline [*r* = −.26]). This indicates that the size of the relations between the FFMQ-15 facets and depression/negative rumination did not differ significantly from the size of relations found between the FFMQ-39 facets and the same constructs, at both pre- and post-MBCT.[Table-anchor tbl2]

### Confirmatory Factor Analyses

Table 3 presents the fit indices for the five CFA models tested for the FFMQ-39 pre-MBCT and post-MBCT, and for the FFMQ-15 pre-MBCT and post-MBCT. For each model, bold indices (CFI, RMSEA, NNFI, and SRMR) indicate that they meet the cutoff criteria for acceptable fit. For both versions of the FFMQ, pre- and post-MBCT, all fit indices indicated that a one-factor model was a poor fit to the data, suggesting that items were not directly subsumed under a unidimensional mindfulness construct. For both versions of the FFMQ, all models fit the data better post-MBCT than pre-MBCT. Based on the fit indices, the four-factor and five-factor models best fit the FFMQ-39 pre-MBCT data, the four-factor and five-factor hierarchical models best fit the FFMQ-15 pre-MBCT data, and the four-factor hierarchical and five-factor models best fit the FFMQ-39 and FFMQ-15 post-MBCT data.[Table-anchor tbl3]

Given the arbitrary nature of the cutoff criteria for fit indices (Schermelleh-Engel et al., 2003), the loadings of items, parcels, or facets onto relevant factors and the relations between facets were also taken into account. Across all four data sets, in a five-factor hierarchical model, all five facets loaded significantly onto an overall mindfulness factor and, in a four-factor hierarchical model, all four facets (minus the observing facet) loaded significantly onto an overarching mindfulness factor (*p*s < .01; see Table 4). Taking these significant loadings into account, this indicates that facets of both versions of the FFMQ at both time points can be considered part of an overall mindfulness factor. All loadings of items and parcels onto relevant facets were also significant (*p*s < .01). However, for the FFMQ-39 pre-MBCT data, the covariance between the observing and nonjudging facets was nonsignificant in a five-factor model (*p* = .43); in a four-factor model, all covariances between facets were significant (*p*s < .01). Additionally, for the FFMQ-15 pre-MBCT data, nonsignificant covariances were found between observing and acting with awareness (*p* = .77), and observing and nonjudging (*p* = .71); in a four-factor model, all covariances were significant (*p*s < .05). All covariances between facets were significant in the post-MBCT models for both versions of the FFMQ (*p*s < .01). This suggests that pre-MBCT, both versions of the FFMQ measure four, not five, related facets of mindfulness (excluding the observing facet).[Table-anchor tbl4]

Table 3 also displays the fit indices for the configural invariance models, which tested the five-factor hierarchical models of the FFMQ-39 or FFMQ-15 before and after MBCT simultaneously. The configural invariance models for both versions of the FFMQ fit poorly to the data; almost all of the indices did not meet the cutoff criteria for acceptable fit. Because configural invariance was not supported, we did not apply further model restrictions to test for weak, strong, or strict factorial invariance.

Taken together, the pattern of findings suggests that a four-factor hierarchical model provided the optimal fit for both versions of the FFMQ pre-MBCT, whereas a five-factor hierarchical model was superior for both versions of the FFMQ post-MBCT. Tests of configural invariance supported this interpretation, by indicating that the structure of both versions of the FFMQ was not equivalent before and after MBCT. Although the fit indices favor the nonhierarchical models, the arbitrary nature of cutoff criteria for these indices (Schermelleh-Engel et al., 2003) coupled with the consideration of other criteria (e.g., significant loadings of facets onto an overarching mindfulness factor as shown in Table 4) lend strong support for the hierarchical models.

## Discussion

The primary aim of this study was to examine the factor structure of the long (39-item) and short (15-item) versions of the FFMQ before and after MBCT, to determine whether the structure remains stable over a period during which people are learning mindfulness meditation. The secondary aim was to assess the general psychometric properties of the FFMQ-39 and FFMQ-15, specifically their sensitivity to change and convergent validity before and after MBCT. We found both versions to be sensitive to change; small/moderate to moderate/large significant increases from pre- to post-MBCT were found for total facet scores from both versions. Additionally, large correlations were found between the total facet scores of the FFMQ-15 and FFMQ-39 (*r* range: .85−.94, *r*_c_ range: .70−.85), indicating that both versions measure highly similar constructs. Convergent validity was tested by correlating FFMQ total facet scores with theoretically related constructs (depression and negative rumination) before and after MBCT. Significant negative correlations were found between rumination/depression and facets of both versions of the FFMQ. Differences in the correlation coefficients between the two versions of the FFMQ were also found to be nonsignificant; the size of the relations between the FFMQ-15 facets and depression/rumination did not differ significantly from the size of relations found between the FFMQ-39 facets and these variables.

Separate CFAs showed that a four-factor hierarchical model, without the observing facet, provided the best fit for both versions of the FFMQ pre-MBCT, whereas a five-factor hierarchical model was superior for both versions of the FFMQ post-MBCT. This was informed by nonsignificant covariances between observing and other facets (nonjudging, acting with awareness) in the FFMQ-39 and FFMQ-15 pre-MBCT models, which were significant in the post-MBCT models. Significant loadings of the facets to a hierarchical latent mindfulness construct also contributed to this interpretation. Additionally, configural invariance was not supported for both versions of the FFMQ; a single model of the five-factor hierarchical structure before and after MBCT corresponded poorly to the data. Taken together, this indicates that the FFMQ’s structural configuration, or the number of factors and pattern of factor loadings, was not equivalent across the two time points.

Our CFA findings support the emerging body of literature that has shown the five-factor hierarchical structure of the FFMQ holds in samples of meditators or people who have undertaken an MBI, but a four-factor hierarchical model best represents data from people with little or no meditation experience (Baer et al., 2006; Baer et al., 2008; Curtiss & Klemanski, 2014a; M. J. Williams et al., 2014). Additionally, the nonsignificant covariances found between observing and nonjudging (for the FFMQ-39 and FFMQ-15) and observing and acting with awareness (for the FFMQ-15) at baseline reflect the nonsignificant relations between observing and other facets found in previous studies (Baer et al., 2006; Bohlmeijer et al., 2011; Curtiss & Klemanski, 2014a). Importantly, our results also extend previous research, by demonstrating that the factor structure of the FFMQ varies before and after MBCT as well as across samples of meditators and nonmeditators. Furthermore, our findings highlight that the FFMQ-39 and FFMQ-15 are both sensitive to change, are consistent in terms of factor structure before and after MBCT, and do not differ significantly from each other with regard to convergent validity.

In relation to the Observing subscale, current findings support the perspective that meditation experience alters people’s qualities of noticing, by enhancing the strength of the relation between observing experience and other aspects of mindfulness (in particular acting with awareness and nonjudging; Baer et al., 2008). It is possible that participants with little or no meditation experience report how much they tend to observe, but the way in which they observe may not be consistent with mindfulness and may instead involve neutral or maladaptive forms of attention. With meditation experience and familiarity with a more accepting and curious way of noticing all experience, not only may people report greater levels of observing, but also the way in which they observe may be more consistent with acting with awareness and nonjudging. For example, observing a negative thought such as “this happy moment won’t last” may be associated with noting and letting the thought pass, while reorienting attention back to the present moment and to other dimensions of experience.

While current findings indicate that people’s quality of observing differs before and after MBCT, they do not provide direct support for the explanation that observing may involve pathologic forms of attentional monitoring pre-MBCT and accepting, curious, and purposeful attention post-MBCT. To test this, studies would need to examine whether the relation between pre-MBCT scores on the Observing subscale and anxious monitoring is significantly greater than the correlation between post-MBCT observing scores and anxious monitoring. Alternatively, studies could compare pre-MBCT observing scores between nonmeditators with a diagnosis of an anxiety disorder, who have a higher degree of anxious monitoring, and nonmeditators in the nonclinical population or without a diagnosis of anxiety. Once a better understanding is reached on how the Observing subscale operates, it may be useful to explore the effects of particular facets (e.g., acting with awareness) on the functioning of the observing facet (e.g., Desrosiers, Vine, Curtiss, & Klemanski, 2014).

### Implications

Several implications arise from our findings for studies investigating change in trait mindfulness, as measured by the FFMQ, before and after mindfulness interventions. Our findings show that total FFMQ scores and scores on the observing facet are not valid for evaluating change from pre to post intervention; pre to post differences in scores on the Observing subscale may reflect changes in the extent to which people notice experience, rather than a genuine change in the ability to observe mindfully. To evaluate interventions that involve mindfulness meditation practice, researchers should consider only comparing the Describing, Acting with Awareness, Nonjudging of Inner Experience, and Nonreactivity to Inner Experience facets and combine only these four subscale scores into a total FFMQ score. Although the empirical evidence suggests that, for nonmeditators, the observing facet does not converge well with other facets that underlie mindfulness, this does not mean that, theoretically, observing experience is not an integral aspect of a mindful disposition. Rather, this suggests that, although the current observing items may reflect how much people tend to notice, they may need revision to better capture the accepting, curious, and purposeful qualities of noticing all experience consistent with a mindful disposition. Future research should also consider using a triangulated approach, whereby alternative methods of measuring mindfulness (e.g., neuropsychological, cognitive, and qualitative measures; see Sauer et al., 2013, for a review) are used to complement the FFMQ.

Additionally, current findings support the FFMQ-15 as a valid and reliable alternative measure to the original FFMQ for use in studies administering multiple measures and/or questionnaires at multiple time points. Furthermore, the significant loadings of all FFMQ-39 and FFMQ-15 facets onto an overall mindfulness factor pre- and post-MBCT support the legitimacy of using a total FFMQ score, alongside total facet scores, as an indicator of global mindfulness level (but without the observing facet in nonmeditating samples). Support for the hierarchical models of the FFMQ also reinforces the theoretical conceptualization of mindfulness as a multifaceted yet coherent construct.

### Limitations and Future Directions

Current findings inform our use of the FFMQ to measure mindfulness, which is essential to advancing research in this area. However, there are several limitations. Prior mediation experience was not measured in the current sample. Participants may have had experience of mindfulness meditation prior to the MBCT program, which would question the validity of attributing changes in the factor structure of the FFMQ to learning mindfulness. It is possible that changes in the factor structure might occur across other types of psychological intervention, following changes in level of depression within the sample, or as a result of the passage of time and retesting. However, our findings showing that a four-factor hierarchical model and a five-factor hierarchical model best fit the data pre-MBCT and post-MBCT, respectively, support previous research conducted in nonmeditator and mediator samples (Baer et al., 2006; Baer et al., 2008; Curtiss & Klemanski, 2014a; M. J. Williams et al., 2014). We also found nonsignificant covariances between observing and nonjudging and acting with awareness facets, which correspond closely with previous findings (Baer et al., 2006; Bohlmeijer et al., 2011; Curtiss & Klemanski, 2014a). These parallels suggest that our sample is likely to have had a similar level of meditation experience pre-MBCT as nonmeditator samples. Nonetheless, meditation status should be recorded in future studies because doing so will allow replication of previous research, by conducting multiple group CFAs to assess whether baseline FFMQ factor structure is altered by meditation experience.

The current sample was also limited to adults with MDD in remission, the population for whom MBCT was originally developed. The present findings should be extended by testing the FFMQ models on pre- and post-MBCT data from other clinical and nonclinical (e.g., students, community samples) samples. We would expect our findings to be replicated in independent samples, provided that participants have little or no meditation experience before MBCT. The factor structure and psychometric properties of the FFMQ-15 should also be tested in additional samples to further support its use. In line with methodological guidelines for the development and validation of short-form measures (Smith, McCarthy, & Anderson, 2000), we recommend that studies conducting additional psychometric testing of the FFMQ-15 administer the short form in its own right to an independent sample (i.e., not a sample in which the FFMQ-39 was administered). In the current study, data for both long and short versions of the FFMQ were based on a single administration of the measure and correlation coefficients were corrected to account for overlapping error variance. However, we recommend that future research examining the overlap of the FFMQ-15 and FFMQ-39 administer both versions, independently, to the same participants.

A further limitation of this study pertains to its sample size (*N* = 238). In the most complex separate CFA model (five-factor correlated model), there were 40 free parameters and, in the configural invariance models, there were 86 free parameters. The common rule of thumb of at least five participants per free parameter (Bentler & Chou, 1987) would mean that the current sample size may have been adequate for separate CFA analyses but not for analyses of configural invariance. However, it is widely acknowledged that rules of thumb for determining sample size requirements do not apply to all situations and need to take into account additional factors, such as degrees of factor overdetermination and item communalities (Meade & Bauer, 2007). Nevertheless, it would be desirable for future studies to replicate the configural invariance analyses using larger clinical and nonclinical samples.

Moreover, future research could assess whether current findings are replicated using other interventions that involve substantial mindfulness meditation practice, such as MBSR, and interventions that include mindfulness principles but less or no meditation practice, such as acceptance and commitment therapy (S. C. Hayes & Wilson, 1994) and briefer self-help MBIs (see Cavanagh, Strauss, Forder, & Jones, 2014, for a review). This could potentially yield interesting insights into the degree to which meditation practice is needed to alter the way we observe experience and whether changes in the factor structure of the FFMQ are caused specifically by meditation practice, or by other factors (e.g., intellectual understanding of mindfulness).

### Conclusion

The FFMQ is a widely used measure of dispositional mindfulness in studies investigating change before and after MBIs, such as MBCT and MBSR. However, our findings show that the factor structure of the FFMQ is not invariant before and after MBCT and findings suggest that researchers should consider omitting the Observing subscale when comparing total scale and subscale scores before and after mindfulness interventions. Current findings also provide initial support for the 15-item version of the FFMQ as a reliable and valid alternative measure for use in studies administering multiple measures and/or measures at multiple occasions.

## Supplementary Material

10.1037/pas0000263.supp

## Figures and Tables

**Table 1 tbl1:** Descriptive Statistics, Reliability Coefficients, and Sensitivity to Change Statistics for FFMQ-39 and FFMQ-15 Facets Pre- and Post-MBCT (N = 238)

Scale and subscale	Pre-MBCT			Post-MBCT			Sensitivity to change	
	*M*	*SD*	α	*M*	*SD*	α	*t*^a^	*d* [95% CI]^b^
FFMQ-39								
Observing	25.00	5.78	.78	28.32	5.02	.82	−10.14*	−0.61 [−0.74, −0.48]
Describing	26.21	6.36	.88	27.79	6.11	.90	−5.28*	−0.25 [−0.35, −0.16]
Acting with Awareness	24.12	5.29	.84	25.87	4.93	.86	−5.40*	−0.34 [−0.47, −0.21]
Nonjudging of Inner Experience	24.72	6.12	.86	27.71	5.85	.88	−7.56*	−0.50 [−0.63, −0.36]
Nonreactivity to Inner Experience	20.10	4.94	.83	22.70	4.28	.85	−7.80*	−0.56 [−0.71, −0.41]
FFMQ-15								
Observing	8.98	2.73	.64	10.37	2.26	.69	−8.78*	−0.55 [−0.68, −0.42]
Describing	9.84	2.74	.80	10.55	2.60	.83	−5.20*	−0.26 [−0.36, −0.16]
Acting with Awareness	9.10	2.25	.68	10.11	2.09	.70	−6.98*	−0.47 [−0.61, −0.33]
Nonjudging of Inner Experience	9.43	2.67	.76	10.71	2.44	.78	−7.31*	−0.50 [−0.64, −0.36]
Nonreactivity to Inner Experience	8.58	2.30	.66	9.68	2.10	.77	−6.47*	−0.50 [−0.65, −0.34]
*Note*. For FFMQ-39 subscales, scores range from 8−40, except for nonreactivity to experience scores, which range from 7−35. For all FFMQ-15 subscales, scores range from 3−15. CI = confidence interval; FFMQ = Five Facet Mindfulness Questionnaire; MBCT = mindfulness-based cognitive therapy.								
^a^ Paired-samples *t* tests comparing pre-MBCT and post-MBCT scores on the FFMQ facets. ^b^ Cohen’s *d* effect sizes, with accompanying 95% CIs.								
* *p* < .001.								

**Table 2 tbl2:** Pearson Correlation Coefficients Between Total Facet Scores on the FFMQ-39 and FFMQ-15 and Other Constructs Pre- and Post-MBCT

	BDI-II		CERTS: Negative Rumination subscale^a^	
Scale and subscale	Pre	Post	Pre	Post
FFMQ-39				
Observing	−.15*	−.18**	−.23**	−.28**
Describing	−.28**	−.29**	−.18*	−.27**
Acting with Awareness	−.38**	−.50**	−.37**	−.53**
Nonjudging of Inner Experience	−.32**	−.44**	−.54**	−.64**
Nonreactivity to Inner Experience	−.24**	−.36**	−.39**	−.49**
FFMQ−15				
Observing	−.15*	−.18**	−.18*	−.23**
Describing	−.25**	−.29**	−.22**	−.28**
Acting with Awareness	−.26**	−.40**	−.35**	−.52**
Nonjudging of Inner Experience	−.30**	−.42**	−.50**	−.58**
Nonreactivity to Inner Experience	−.26**	−.36**	−.36**	−.43**
*Note*. BDI-II = Beck Depression Inventory−II; CERTS = Cambridge Exeter Ruminative Thinking Scale; FFMQ = Five Facet Mindfulness Questionnaire; MBCT = mindfulness-based cognitive therapy.				
^a^ This measure was only completed by participants in the PREVENT trial (*N* = 154). In the PREVENT sample, 46 participants (30%) were male and 108 (70%) female. Mean age was 52.12 years (*SD* = 11.18) and 152 (98.7%) of the sample were white. Ten participants (7%) had no qualifications, 23 (15%) had some General Certificate of Secondary Education/O-Level qualifications, 64 (42%) had A-Levels or vocational qualifications, 37 (24%) had a bachelor’s degree, eight (5%) had a master’s degree, and 10 (6.5%) had a doctoral degree or professional qualification. Data on education were missing for two participants.				
* *p* < .05. ** *p* < .01.				

**Table 3 tbl3:** CFA Fit Indices for the Five Models Tested Pre- and Post-MBCT for Both Long (FFMQ-39) and Short (FFMQ-15) Versions of the FFMQ and the Configural Invariance Models

Model	CFI	RMSEA [90% CI]	NNFI	SRMR	χ^2^	*df*	AIC
FFMQ-39 pre-MBCT							
One-factor	.455	.225 [.213, .236]	.364	.168	1,167.386	90	1,227.386
Four-factor	**.950**	**.086 [.069, .103]**	.931	**.052**	131.792	48	191.792
Four-factor hierarchical^a^	.939	**.092 [.076, .110]**	.920	**.071**	151.358	50	207.358
Five-factor	**.951**	**.071 [.057, .085]**	.936	**.050**	175.948	80	255.948
Five-factor hierarchical^b^	.932	**.082 [.069, .095]**	.916	**.085**	219.807	85	289.807
FFMQ-39 post-MBCT							
One-factor	.508	.230 [.219, .242]	.426	.142	1,222.433	90	1,282.433
Four-factor	**.987**	**.047 [.023, .068]**	**.982**	**.037**	72.908	48	132.908
Four-factor hierarchical	**.986**	**.047 [.024, .068]**	**.981**	**.042**	76.453	50	132.453
Five-factor	**.987**	**.040 [.018, .057]**	**.983**	**.037**	110.068	80	190.068
Five-factor hierarchical	**.969**	**.060 [.045, .074]**	**.961**	**.068**	157.012	85	227.012
FFMQ-15 pre-MBCT							
One-factor	.458	.157 [.145, .169]	.367	.127	613.755	90	673.755
Four-factor	.932	**.070 [.052, .089]**	.906	**.076**	103.992	48	163.992
Four-factor hierarchical	.915	**.077 [.059, .094]**	.888	**.084**	119.699	50	175.699
Five-factor	.925	**.062 [.047, .077]**	.902	**.071**	152.158	80	232.158
Five-factor hierarchical	.895	**.071 [.057, .085]**	.870	**.089**	186.794	85	256.794
FFMQ-15 post-MBCT							
One-factor	.553	.159 [.147, .171]	.478	.118	627.361	90	687.361
Four-factor	**.956**	**.062 [.042, .081]**	.940	**.058**	91.676	48	151.676
Four-factor hierarchical	**.956**	**.061 [.042, .080]**	.942	**.060**	94.213	50	150.213
Five-factor	**.947**	**.058 [.042, .073]**	.930	**.055**	143.624	80	223.624
Five-factor hierarchical	.926	**.067 [.052, .081]**	.908	**.069**	174.442	85	244.442
FFMQ-39 configural invariance^c^	.886	**.079 [.073, .085]**	.869	.104	941.057	379	1,113.057
FFMQ-15 configural invariance	.825	**.073 [.066, .079]**	.800	.096	852.401	379	1,024.401
*Note.* Indices in boldface fall within the acceptable range when rounded up or down to two decimal places. AIC = Akaike information criterion; CFI = comparative fit index; CI = confidence interval; FFMQ = Five Facet Mindfulness Questionnaire; MBCT = mindfulness-based cognitive therapy; NNFI = non-normed fit index; RMSEA = root mean square error of approximation; SRMR = standardized root mean square residual.							
^a^ Four-factor hierarchical refers to the model in which the facets acting with awareness, nonjudging, and nonreactivity (without the observing facet) were loaded onto an overall mindfulness factor. ^b^ Five-factor hierarchical refers to the model in which all five facets were loaded onto an overall mindfulness factor. ^c^ Configural invariance refers to the model in which pre- and post-MBCT five-factor hierarchical models were tested simultaneously. One model was tested for the FFMQ-39 and one for the FFMQ-15.							
* *p* < .001.							

**Table 4 tbl4:** Standardized Loadings of the Facets Onto an Overarching Mindfulness Factor for the FFMQ-39 and FFMQ-15 Four-Factor Hierarchical and Five-Factor Hierarchical Models Pre-MBCT and Post-MBCT

Path	Pre-MBCT	Post-MBCT
FFMQ-39 four-factor hierarchical		
Describing ← Mindfulness	.63	.57
Acting with Awareness ← Mindfulness	.72	.75
Nonjudging ← Mindfulness	.53	.74
Nonreactivity ← Mindfulness	.63	.73
FFMQ-39 five-factor hierarchical		
Observing ← Mindfulness	.58	.63
Describing ← Mindfulness	.71	.59
Acting with Awareness ← Mindfulness	.56	.65
Nonjudging ← Mindfulness	.39	.66
Nonreactivity ← Mindfulness	.75	.86
FFMQ-15 four-factor hierarchical		
Describing ← Mindfulness	.41	.47
Acting with Awareness ← Mindfulness	.59	.69
Nonjudging ← Mindfulness	.73	.86
Nonreactivity ← Mindfulness	.58	.60
FFMQ-15 five-factor hierarchical		
Observing ← Mindfulness	.35	.55
Describing ← Mindfulness	.54	.51
Acting with Awareness ← Mindfulness	.43	.65
Nonjudging ← Mindfulness	.53	.72
Nonreactivity ← Mindfulness	.81	.73
*Note.* All loadings of facets onto an overarching mindfulness factor were significant (*p*s < .01). FFMQ = Five Facet Mindfulness Questionnaire; MBCT = mindfulness-based cognitive therapy.		
