# Supplementary material for: Examining the Factor Structure of the 39-Item and 15-Item Versions of the Five Facet Mindfulness Questionnaire Before and After Mindfulness-Based Cognitive Therapy for People With Recurrent Depression
Source: Psychol Assess. 2016 Apr 14;28(7):791–802. doi: 10.1037/pas0000263 (PMC4928699; doi:10.1037/pas0000263)
Supplement: Supplementary file 1 [file z1t004153027st1.doc]

**Supplemental Materials**

**Examining the Factor Structure of the 39-Item and 15-Item Versions of the Five-Facet Mindfulness Questionnaire Before and After Mindfulness-Based Cognitive Therapy for People With Recurrent Depression**

**by J. Gu et al., 2016, *Psychological Assessment***

**http://dx.doi.org/10.1037/pas0000263**

Table S1

*Unstandardized and Standardized Loadings for the FFMQ-39 Four-Factor Hierarchical ^a^ Pre-MBCT Model*

| Path | Unstandardized estimate (*SE*) | Standardized estimate |
| --- | --- | --- |
| Factor loadings |  |  |
| Describing <- Mindfulness | 1.31 (.24)*** | .63 |
| Acting with awareness <- Mindfulness | 1.38 (.25)*** | .72 |
| Nonjudging <- Mindfulness | 1.05 (.21)*** | .53 |
| Nonreactivity <- Mindfulness | 1.00 | .63 |
| Item loadings |  |  |
| Parcel 1 <- Acting with awareness | 1.00 | .89 |
| Parcel 2 <- Acting with awareness | 0.91 (.06)*** | .84 |
| Parcel 3 <- Acting with awareness | 0.83 (.06)*** | .75 |
| Parcel 1 <- Nonjudging | 1.00 | .85 |
| Parcel 2 <- Nonjudging | 0.92 (.07)*** | .80 |
| Parcel 3 <- Nonjudging | 1.14 (.09)*** | .80 |
| Parcel 1 <- Describing | 1.00 | .87 |
| Parcel 2 <- Describing | 0.98 (.06)*** | .87 |
| Parcel 3 <- Describing | 1.19 (.06)*** | .90 |
| Parcel 1 <- Nonreactivity | 1.00 | .77 |
| Parcel 2 <- Nonreactivity | 1.20 (.11)*** | .78 |
| Parcel 3 <- Nonreactivity | 1.33 (.11)*** | .84 |

*Note.* FFMQ = Five-Facet Mindfulness Questionnaire; MBCT = Mindfulness-Based Cognitive Therapy; SE = standard error.

^a^ Four-factor hierarchical refers to the model in which the facets describe, acting with awareness, nonjudging, and nonreactivity (without the observing facet) loaded on to an overall mindfulness factor.

****p* < .001.

Table S2

*Unstandardized and Standardized Loadings for the FFMQ-39 Five-Factor Hierarchical* ^a^ *Post-MBCT Model*

| Path | Unstandardized estimate (*SE*) | Standardized estimate |
| --- | --- | --- |
| Factor loadings |  |  |
| Observing <- Mindfulness | 0.77 (.11)*** | .63 |
| Describing <- Mindfulness | 0.94 (.13)*** | .59 |
| Acting with awareness <- Mindfulness | 0.85 (.11)*** | .65 |
| Nonjudging <- Mindfulness | 0.92 (.12)*** | .66 |
| Nonreactivity <- Mindfulness | 1.00 | .86 |
| Item loadings |  |  |
| Parcel 1 <- Observing | 1.00 | .82 |
| Parcel 2 <- Observing | 1.06 (.08)*** | .92 |
| Parcel 3 <- Observing | 0.99 (.09)*** | .67 |
| Parcel 1 <- Describing | 1.00 | .91 |
| Parcel 2 <- Describing | 0.92 (.05)*** | .89 |
| Parcel 3 <- Describing | 1.07 (.05)*** | .90 |
| Parcel 1 <- Nonreactivity | 1.00 | .88 |
| Parcel 2 <- Nonreactivity | 1.03 (.08)*** | .75 |
| Parcel 3 <- Nonreactivity | 1.17 (.08)*** | .82 |
| Parcel 1 <- Nonjudging | 1.00 | .85 |
| Parcel 2 <- Nonjudging | 1.08 (.07)*** | .84 |
| Parcel 3 <- Nonjudging | 1.12 (.08)*** | .83 |
| Parcel 1 <- Acting with awareness | 1.00 | .89 |
| Parcel 2 <- Acting with awareness | 0.92 (.06)*** | .83 |
| Parcel 3 <- Acting with awareness | 0.95 (.06)*** | .84 |

*Note.* FFMQ = Five-Facet Mindfulness Questionnaire; MBCT = Mindfulness-Based Cognitive Therapy; SE = standard error.

^a^ Five-factor hierarchical refers to the model in which all five facets loaded on to an overall mindfulness factor.

****p* < .001.

Table S3

*Unstandardized and Standardized Loadings for the FFMQ-15 Four-Factor Hierarchical ^a^ Pre-MBCT Model*

| Path | Unstandardized estimate (*SE*) | Standardized estimate |
| --- | --- | --- |
| Factor loadings |  |  |
| Describing <- Mindfulness | 0.93 (.28)*** | .41 |
| Acting with awareness <- Mindfulness | 0.56 (.17)*** | .59 |
| Nonjudging <- Mindfulness | 1.64 (.42)*** | .73 |
| Nonreactivity <- Mindfulness | 1.00 | .58 |
| Item loadings |  |  |
| Item #8 <- Acting with awareness | 1.00 | .41 |
| Item #34 <- Acting with awareness | 2.02 (.36)*** | .71 |
| Item #38 <- Acting with awareness | 2.33 (.43)*** | .86 |
| Item #14 <- Nonjudging | 1.00 | .74 |
| Item #10 <- Nonjudging | 0.74 (.09)*** | .62 |
| Item #30 <- Nonjudging | 1.03 (.11)*** | .79 |
| Item #2 <- Describing | 1.00 | .80 |
| Item #16 <- Describing | 0.76 (.08)*** | .64 |
| Item #27 <- Describing | 1.11 (.11)*** | .84 |
| Item #19 <- Nonreactivity | 1.00 | .63 |
| Item #29 <- Nonreactivity | 1.29 (.20)*** | .85 |
| Item #33 <- Nonreactivity | 0.69 (.12)*** | .46 |

*Note.* FFMQ = Five-Facet Mindfulness Questionnaire; MBCT = Mindfulness-Based Cognitive Therapy; SE = standard error.

^a^ Four-factor hierarchical refers to the model in which the facets describe, acting with awareness, nonjudging, and nonreactivity (without the observing facet) loaded on to an overall mindfulness factor.

****p* < .001.

Table S4

*Unstandardized and Standardized Loadings for the FFMQ-15 Five-Factor Hierarchical ^a^ Post-MBCT Model*

| Path | Unstandardized estimate (*SE*) | Standardized estimate |
| --- | --- | --- |
| Factor loadings |  |  |
| Observing <- Mindfulness | 0.72 (.16)*** | .55 |
| Describing <- Mindfulness | 0.86 (.17)*** | .51 |
| Acting with awareness <- Mindfulness | 0.59 (.13)*** | .65 |
| Nonjudging <- Mindfulness | 1.06 (.19)*** | .72 |
| Nonreactivity <- Mindfulness | 1.00 | .73 |
| Item loadings |  |  |
| Item #6 <- Observing | 1.00 | .64 |
| Item #11 <- Observing | 1.00 (.15)*** | .56 |
| Item #15 <- Observing | 1.11 (.16)*** | .78 |
| Item #2 <- Describing | 1.00 | .78 |
| Item #16 <- Describing | 0.95 (.09)*** | .76 |
| Item #27 <- Describing | 1.08 (.10)*** | .81 |
| Item #19 <- Nonreactivity | 1.00 | .71 |
| Item #29 <- Nonreactivity | 1.04 (.11)*** | .82 |
| Item #33 <- Nonreactivity | 0.87 (.10)*** | .68 |
| Item #14 <- Nonjudging | 1.00 | .72 |
| Item #10 <- Nonjudging | 0.95 (.11)*** | .63 |
| Item #30 <- Nonjudging | 1.24 (.12)*** | .89 |
| Item #8 <- Acting with awareness | 1.00 | .49 |
| Item #34 <- Acting with awareness | 1.48 (.23)*** | .70 |
| Item #38 <- Acting with awareness | 1.78 (.28)*** | .84 |

*Note.* FFMQ = Five-Facet Mindfulness Questionnaire; MBCT = Mindfulness-Based Cognitive Therapy; SE = standard error.

^a^ Five-factor hierarchical refers to the model in which all five facets loaded on to an overall mindfulness factor.

****p* < .001.
